# Goose STING mediates IFN signaling activation against RNA viruses

**DOI:** 10.3389/fimmu.2022.921800

**Published:** 2022-07-26

**Authors:** Feiyu Fu, Zhenyu Lin, Yanlin Li, Jie Wang, Yawen Li, Pengcheng Liu, Zhaofei Wang, Jingjiao Ma, Yaxian Yan, Jianhe Sun, Yuqiang Cheng

**Affiliations:** Shanghai Key Laboratory of Veterinary Biotechnology, Key Laboratory of Urban Agriculture (South), Ministry of Agriculture, School of Agriculture and Biology, Shanghai Jiao Tong University, Shanghai, China

**Keywords:** goose, STING, IFN, innate immunity, RNA virus

## Abstract

Stimulator of the interferon gene (STING) is involved in mammalian antiviral innate immunity as an interferon (IFN) activator. However, there is still a lack of clarity regarding the molecular characterization of goose STING (GoSTING) and its role in the innate immune response. In the present study, we cloned GoSTING and performed a series of bioinformatics analyses. GoSTING was grouped into avian clades and showed the highest sequence similarity to duck STING. The *in vitro* experiments showed that the mRNA levels of GoSTING, IFNs, IFN-stimulated genes (ISGs), and proinflammatory cytokines were significantly upregulated in goose embryo fibroblast cells (GEFs) infected with Newcastle disease virus (NDV). Overexpression of GoSTING in DF-1 cells and GEFs strongly activated the IFN-β promoter as detected by a dual-luciferase reporter assay. Furthermore, overexpression of GoSTING induced the expression of other types of IFN, ISGs, and proinflammatory cytokines and inhibited green fluorescent protein (GFP)-tagged NDV (NDV-GFP) and GFP-tagged vesicular stomatitis virus (VSV) (VSV-GFP) replication *in vitro*. In conclusion, these data suggest that GoSTING is an important regulator of the type I IFN pathway and is critical in geese’s innate immune host defense against RNA viruses.

## Introduction

The innate immune system is the host’s first line of defense against foreign pathogen invasion and endogenous damage, which initiates appropriate host defense mechanisms by detecting a series of pathogen-associated molecular patterns (PAMPs) and endogenous danger-associated molecular patterns (DAMPs) through pattern recognition receptors (PRRs) (1, 2). After PRRs are activated, downstream signaling pathways are triggered, resulting in the expression of chemokines, proinflammatory cytokines, and the synthesis of type I interferon (IFN) and type III IFN, which induces the expression of IFN-stimulated genes (ISGs) through IFN receptor and Janus kinase (JAK) - signal transducer and activator of transcription (STAT) signaling, to effectively inhibit the replication of pathogens, remove aliens, maintain the physiological balance of the body, and act as a driving force to influence subsequent adaptive immunity (3–6). Currently, the major families of PRRs include the Toll-like receptors (TLRs), the retinoic acid-inducible gene (RIG)-I-like receptors (RLRs), and the nucleotide oligomerization domain-like receptors (NLRs, also called NACHT, LRR and PYD domain proteins) and cytosolic DNA sensors (7).

In addition to PRRs, several junction adaptor proteins are also critical for the induction of IFN, thereby ensuring the normal function of the innate immune system (8, 9). For example, all TLRs family members associate with corresponding adaptor proteins after sensing PAMPs or DAMPs, such as myeloid differentiation major response gene 88 (MyD88), MyD88-adaptor-like protein (MAL), TIR domain-containing adaptor inducing IFN-β (TRIF/TICAM1), TRIF-related adaptor molecule (TRAM/TICAM2), and ultimately activate downstream nuclear factor-κB (NF-κB), IFN regulatory factors (IRFs) and mitogen-activated protein kinase (MAPK) (10–12). Like MyD88, stimulator of the IFN gene (STING; also known as MITA, MYPS, ERIS, and TMEM173), a molecule in the endoplasmic reticulum (ER), is also a key adapter protein with a potent ability to induce type I IFNs, interleukins and other proinflammatory factors (9, 13, 14).

A large number of recent studies have confirmed that STING is the core molecule of innate immune response from pathogen cytosolic DNA and RNA (15). In the RNA-triggered pathway, STING is mainly involved in RIG-I rather than melanoma differentiation-associated gene 5 (MDA5) signaling and functions downstream of RIG-I and mitochondrial antiviral signaling protein (MAVS) and upstream of TANK-binding kinase 1 (TBK1) (9). Following the recognition of RNA ligands, RIG-I is activated by the virus and transmits the signal to MAVS located downstream of the mitochondria, where it interacts with STING. STING is then transported to the vesicle structure around the nucleus and acts as a reaction platform to recruit TBK1 to activate IFN regulator factor (IRF) 3, which forms a dimer and enters the nucleus to induce the synthesis of IFN (16). During the recognition of DNA viruses, the DNA sensors, such as DAI (17), IFI16 (18), DDX41 (19), and cGAS (20, 21), transmit the signal to STING after recognizing DNA, which then activates the IFN *via* the STING-TBK1-IRF3 pathway (8). IFI16 is mainly located in the nucleus and can recognize single-stranded DNA (ssDNA) or double-stranded DNA (dsDNA) ligands in a length-dependent manner through its HIN domain (22). After binding to the ligand, IFI16 induces the expression of IFN-β through the activation of IRF3, and this expression is STING-dependent (23). DDX41 was identified as a cytoplasmic sensor capable of recognizing viral dsDNA. After sensing dsDNA through its DEADc domain, DDX41 binds to STING and initiates activation of the IFN pathway (19, 22). It can be seen that although the localization of various DNA sensors in cells, the nucleic acid forms recognized, and the site that binds to viral nucleic acids are different, the process involves STING as a key mediator. Overall, STING plays an important role in antiviral innate immunity as a key signaling molecule that regulates type I IFNs, which is essential for establishing antiviral status.

Most of the studies mentioned above on STING have focused on mammals, whereas STING signaling events in geese have not been studied. Geese, like ducks, belong to waterfowl and play a critical role in the transmission and dissemination of many important pathogens (14). In particular, Newcastle disease virus (NDV) and avian influenza virus (AIV) cause serious and economically significant diseases in almost all birds (24, 25). Chickens are susceptible to NDV and AIV due to a lack of RIG-I naturally. Instead, chickens express MDA5 or other as yet unidentified receptors which functionally compensate for the absence of RIG-I in the chicken genome (26–28). In addition, preliminary research in our laboratory demonstrated that chicken STING (chSTING) inhibited the replication of NDV and AIV and activated IRF-7 and NF-κB to induce the production of type I IFNs, possibly by participating in the MDA5-STING-IFN-β signaling pathway in chicken cells (29). Compared to chickens lacking RIG-I, ducks and geese encode RIG-I with a similar domain organization to mammals and are generally resistant to NDV and AIV (30). Overexpression of duck STING (DuSTING) has been shown to activate the type I IFN pathway and limit the replication of H9N2 AIV in our previous studies (31).

Interestingly, compared with duck RIG-I, goose RIG-I (GoRIG-I) exhibited a higher IFN-activating ability in DF-1 cells infected with or not infected with the influenza virus (30). Ding et al. have identified the key role of GoRIG-I in innate immunity against NDV infection, and goose MAVS was identified as a GoRIG-I interactive protein involved in the activation of type I IFN pathways goose cells (26, 32). However, whether goose STING (GoSTING), like its mammalian counterpart, also induces type I IFN signaling and exerts antiviral effects remains unclear.

In the present study, we cloned GoSTING and explored the function of GoSTING in innate immunity in geese. We investigated the function of GoSTING in RNA virus infection, and the effects of GoSTING on the inhibition of viral genomic RNA replication based on NDV infection were characterized *in vitro*. Furthermore, our results suggest that GoSTING is an important regulator of IFNs, proinflammatory cytokines, and ISGs in geese. These findings contribute to a more systematic understanding of the bird’s biological role of STING in the innate immune system and provide new insight into general and individual characteristics of the innate immune system in birds and mammals.

## Materials and methods

### Cells and viruses

DF-1 is a chicken embryonic fibroblast cell line from East Lansing strain eggs (33). Goose embryo fibroblast cells (GEFs) were prepared from 15-day-old goose embryos. The DF-1 cells and GEFs were maintained in high-glucose complete Dulbecco’s Modified Eagle’s Medium (DMEM; Corning, USA) supplemented with 10% fetal bovine serum (FBS; Nulen, Shanghai, China) and 1% penicillin-streptomycin (Gibco, USA). All cells were incubated at 37°C in a 5% CO_2_ incubator. The NDV strain NSD14 was isolated from chickens at a farm in Shandong Province, China. Green fluorescent protein (GFP) tagged NDV low virulent strain LaSota named NDV-GFP, and GFP tagged vesicular stomatitis virus (VSV) VSV-GFP were stored in our laboratory. These viruses were purified, propagated, and stored as described in our previous study (29).

### Cloning and bioinformatics analysis of GoSTING

Based on the predicted GoSTING sequence from the National Center for Biotechnology Information (NCBI), the primers GoSTING-F and GoSTING-R (**Table 1**), which were located outside of the GoSTING open reading frame (ORF), were designed to amplify potential GoSTING cDNA *via* RT-PCR on total RNA extracted from the GEF cells. The PCR product was ligated into a pTOPO-Blunt vector (Aidlab Biotech, Beijing, China) for sequencing, and the positive colonies were sent to the Beijing Genomics Institute (Beijing, China) for sequencing. The deduced amino acid sequence of GoSTING was analyzed using the SMART program. The amino acid sequence of GoSTING was aligned with the other animal STING proteins from ducks, chickens, humans, and pigs using Clustal W and edited with ESPript 3.0 (http://http://espript.ibcp.fr/ESPript/cgi-bin/ESPript.cgi). Sequence homology and phylogenetic analysis of amino acid sequences was constructed using DNASTAR software. A phylogenetic tree was constructed based on the STING from 13 different species, including mammals, birds, and fish. Homology modeling for GoSTING was conducted using the online protein-modeling server SWISS-MODEL (http://swissmodel.expasy.org/).

**Table 1:** PCR primers used in this study.

Target Gene		Purpose	Name	Sequence of Oligonucleotide (5’–3’)
**GoIFN-α**		qRT-PCR	qGoIFN-α F	CTCCAGCACCTCTTCGACAC
			qGoIFN-α R	GTTGATGCCGAGGTGAAGGT
**GoIFN-γ**		qRT-PCR	qGoIFN-γ F	ACATCAAAAACCTGTCTGAGCAGC
			qGoIFN-γ R	AGGTTTGACAGGTCCACGAGG
**GoIFN-κ**		qRT-PCR	qGoIFN-κ F	ACAGCAAAGAAAAGTGATTG
			qGoIFN-κ R	GTTGGAAGATCTCTTCAATGG
**GoIFN-λ**		qRT-PCR	qGoIFN-λ F	GAGCTCTCGGTGCCCGACC
			qGoIFN-λ R	CTCAGCGGCCACGCAGCCT
**GoIL-6**		qRT-PCR	qGoIL-6 F	AGCAAAAAGTTGAGTCGCTGTGC
			qGoIL-6 R	TAGCGAACAGCCCTCACGGT
**GoIL-8**		qRT-PCR	qGoIL-8 F	GCTGTCCTGGCTCTTCTCCTGATT
			qGoIL-8 R	GGGTCCAAGCACACCTCTCTGTTG
**GoPKR**		qRT-PCR	qGoPKR F	GCAACAGCAAAGACTGACGA
			qGoPKR R	TGTTTGTGACCTCTGCCTTG
**GoOASL**		qRT-PCR	qGoOASL F	CAGCGTGTGGTGGTTCTC
			qGoOASL R	AACCAGACGATGACATACAC
**GoMx-1**		qRT-PCR	qGoMx-1 F	TTCACAGCAATGGAAAGGGA
			qGoMx-1 R	ATTAGTGTCGGGTCTGGGA
**GoSTING**		qRT-PCR	qGoSTING F	CCATGTCTCAGGACGAGTGC
			qGoSTING R	TCCTCGTATGCAATGAGCCG
		To obtain sequence	GoSTING F	ATGTCTCAGGAACCGCAGCGC
			GoSTING R	CTGCGGAGCGACCACCCCTGA
		Construction of GoSTING	pcDNA3.1-Flag EcoR I	TAGTCCAGTGTGGTGGAATTCATGTCTCAGGAACCGCAGCGC
			pcDNA3.1-Flag Xho I	GTCGTCCTTGTAGTCCTCGAGCTGCGGAGCGACCACCCCTGA
	Construct truncated forms of GoSTING		GoSTING d1-50 aa F	GTGTGGTGGAATTCATG CACCGCCTCACCGCC
			GoSTING d1-50 aa R	CATGAATTCCACCACAC
			GoSTING d1-150 aa F	GTGTGGTGGAATTCATG ACTGAGAGGTCCAAG
			GoSTING d1-150 aa R	CATGAATTCCACCACAC
			GoSTING d50-340 aa F	AGCCCCTGTCACCCGCT CAGGAGGAGTTCACG
			GoSTING d50-340 aa R	AGCGGGTGACAGGGGCT
			GoSTING d181-382 aa F	TGCCACGCATAAAGGAG CTCGAGGACTACAAG
			GoSTING d181-382 aa R	CTCCTTTATGCGTGGCA
			GoSTING d251-382 aa F	ACAGCTTCTACGCAATC CTCGAGGACTACAAG
			GoSTING d251-382 aa R	GATTGCGTAGAAGCTGT
			GoSTING d351-382 aa F	CGGTGTACGAGGGGACC CTCGAGGACTACAAG
			GoSTING d351-382 aa R	GGTCCCCTCGTACACCG
			GoSTING d365-371 aa F	TGGGCTCAACAGACCTC GACCTGCCCCAGCCC
			GoSTING d374-382 aa F	TCAGTGCCTCCGACCTG CTCGAGGACTACAAG
			GoSTING d379-382 aa R	CCGCAGGGGCTGGGGCA
			GoSTING S369A F	CTCAGCCTCCAGATCGCTGCCTCCGACCT
			GoSTING S369A R	GCGATCTGGAGGCTGAGGTCT

### Construction of plasmids

The PCR primers are shown in **Table 1**. The expression construct pcDNA3.1-GoSTING-Flag was constructed by inserting full-length GoSTING into the *Xho* I and *Eco*R I sites of the pcDNA3.1-Flag expression vector *via* homologous recombination. The chicken IFN-β (ch-IFN-β) promoter-luciferase reporter plasmids pGL-IFN-β-Luc was constructed from chick embryo fibroblast genomic DNA using primers with *Nhe* I and *Bgl* II sites (IFN-β-P F and IFN-β-P R) to amplify -158 to +14 of the chicken IFN-β promoter motif, as described previously (29). The promoter fragment was inserted between *Nhe* I and *Bgl* II sites of the pGL3-basic luciferase reporter vector. The truncated plasmids of GoSTING, including d1-50 aa, d1-150 aa, d50-340 aa, d181-382 aa, d251-382 aa, d251-382 aa, d351-382 aa, d365-371 aa, d374-382 aa, d379-382 aa, and S369A were constructed using a modified homologous recombination method and the primers listed in **Table 1**.

### Luciferase reporter assays

The DF-1 or GEF cells were plated in 24-well plates (NEST Biotechnology, Wuxi, China) and transiently transfected with the reporter plasmid pGL-IFN-β-Luc (0.12 μg/well) and internal control Renilla luciferase (PRL-TK, 0.06 μg/well) along with the indicated plasmids using Nulen PlusTrans™ Transfection Reagent (Nulen, Shanghai, China). According to the manufacturer’s instructions, the cells were lysed 24 hours after transfection, and luciferase activity was measured using a Dual-Luciferase Reporter Assay System kit (Promega, USA). Renilla luciferase activity was employed for normalization. All reporter assays were repeated at least three times.

### Reverse transcription-quantitative real-time PCR

RNA was extracted from GEFs using an HP Total RNA kit (Omega, USA), and then the RNA was reverse-transcribed to cDNA using a cDNA synthesis kit (Vazyme). Reverse transcription-quantitative real-time PCR (qRT-PCR) tests were conducted according to the manufacturer’s instructions using a ChamQTM SYBR^®^ qPCR Master Mix (Vazyme). The conditions and data processing method for the qRT-PCR test were previously described (29).

### Virus infection and qRT-PCR analysis

For antiviral effect evaluation, GEF cells were transfected with pcDNA3.1-GoSTING-Flag plasmid or empty plasmid. After 24 hours, the GEF cells were washed twice with PBS (Gibco) and infected at 0.05 multiplicity of infection (MOI) with NSD14. The RNA from the cells, which were infected with the viruses at different times, was then collected for qRT-PCR to measure the mRNA level of GoSTING. The GoSTING-overexpressing and normal DF-1 cells were infected at 0.01 MOI with NDV-GFP or VSV-GFP, and fluorescence was measured 24h after infection using a fluorescence microscope.

### Western blot analysis

The DF-1 cells were plated in 12-well plates at 1×10^6^/mL and then transfected with a total of empty plasmid or GoSTING expression plasmid. At thirty-six hours post-transfection, cells were washed twice with phosphate buffer saline (PBS) (Gibco) and then lysed with a cell lysis buffer (Beyotime, Shanghai, China) containing an InStab™ protease cocktail (Yeasen, Shanghai, China) and phenylmethylsulfonyl fluoride (PMSF) (Yeasen). Lysates were centrifuged at 13,000 rpm for 15 minutes to obtain the supernatant and were eluted with a 5×SDS-PAGE loading buffer (Yeasen) and boiled for 10 min. Then the cell lysates were separated *via* SDS-PAGE and analyzed by Western blotting. Images were collected with the Tanon 5200 imaging system (Tanon, Shanghai, China), as described in our previous study (34).

### Statistical analysis

Data were expressed as means ± standard deviations, with three biological replicates for each experiment. The two-tailed independent Student’s t-test was used to determine the significance. (*P < 0.05, ** *P* < 0.01, *** *P* < 0.001, **** *P* < 0.0001).

### Ethics statements

The studies involving goose embryos were conducted in the laboratory of Shanghai Veterinary Research Institute. The studies were reviewed and approved by the Animal Ethics Committee of Shanghai Veterinary Research Institute (20210521).

## Results

### Cloning and sequence analysis of GoSTING

Based on a predicted goose sequence (XM_013202032.1) from NCBI, primers GoSTING-F and GoSTING-R (**Table 1**), located outside of GoSTING ORF, were designed and used to amplify potential GoSTING cDNA using RT-PCR on total RNA extracted from the GEF cells.

**Figure 1:**
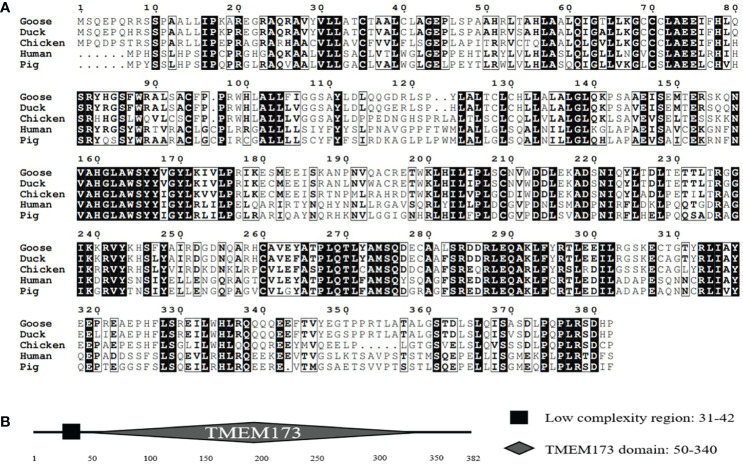
**(A)** The alignment of the deduced amino acid sequence of GoSTING with other animal STING proteins from the ducks, chickens, humans, and pigs was performed using the Clustal W program and edited with ESPript 3.0. The black shading indicates the identity of the amino acid, and the gray shading indicates similarity. (50% threshold). **(B)** The prediction of protein domains of GoSTING.

Based on cDNA, the full-length GoSTING gene contains 1149 bp and encodes 382 amino acid (aa) residues (**Figure 1A**). Multiple sequence alignment showed that the amino acid sequences of GoSTING are 93.5, 44.0, and 61.8% identical to the STING gene in ducks (XP_027311055.1), humans (NP_938023.1), zebra finches (NP_001232785.1), respectively. The protein domains of GoSTING were predicted using the SMART program. The results show that GoSTING consists of a low compositional complexity region (31-42aa) and the TMEM173 (50-340aa) domain (**Figure 1B**).

### Phylogenetic tree analyses and the three-dimensional structure of GoSTING

**Figure 2:**
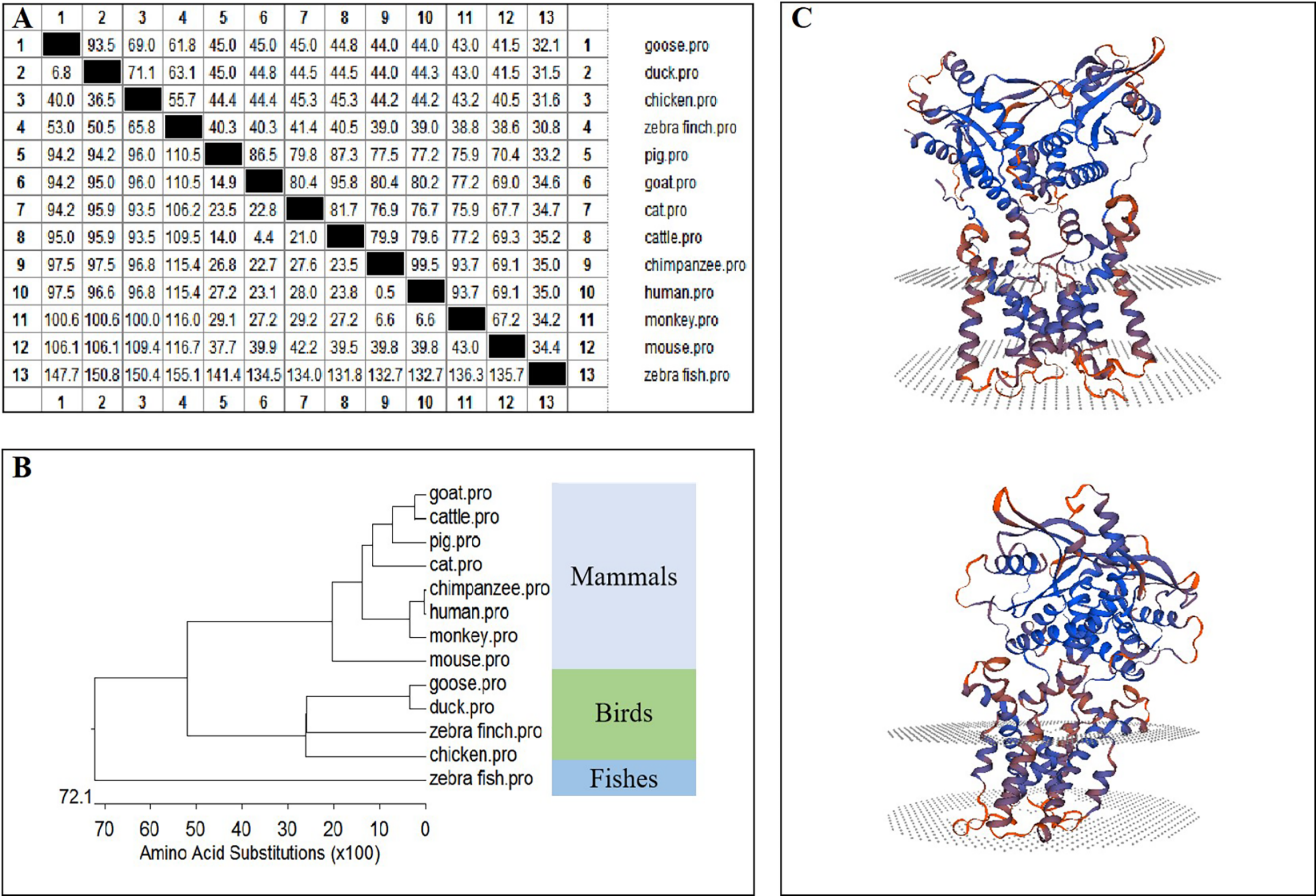
**(A)** The amino acid sequence homology of different animals. **(B)** Phylogenetic tree of the deduced amino acid sequence of GoSTING and other animal STING proteins. **(C)** Three-dimensional structure of GoSTING predicted by SWISS-MODEL.

The amino acid sequence homologies of different animals were conducted using MegAlign, and the results are shown in **Figure 2A**. A phylogenetic tree was developed based on multiple alignments of STING from various species, including fish, birds, and mammals. Phylogenetic analysis showed that the goose, duck, zebra finch, and chicken STING protein sequences were in the same subgroup. STING from mammals, including goats, cattle, pigs, cats, chimpanzees, humans, monkeys, and mice, was in another subgroup, and fish STING was in a third subgroup (**Figure 2B**). The predicted three-dimensional structures of GoSTING are shown in **Figure 2C**.

### Upregulation of GoSTING expression during viral infection

**Figure 3:**
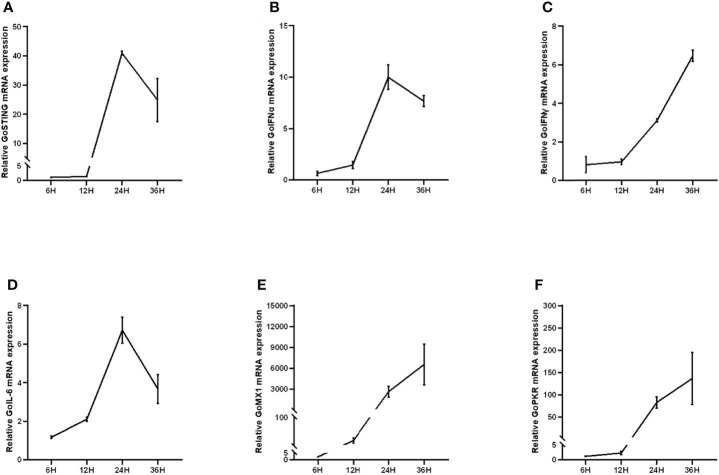
**(A)** Upregulation of GoSTING in GEF cells infected with NDV at 0.05 MOI. **(B, C)** Upregulation of IFNs (IFN-α and IFN-γ) in GEFs infected with NDV at 0.05 MOI. **(D)** Upregulation of IL-6 in GEFs infected with NDV at 0.05 MOI. **(E, F)** Upregulation of ISGs (Mx-1, PKR) in GEFs infected with NDV at 0.05 MOI. Error bars represent standard deviations.

In mammals, STING is involved in the type I IFN-mediated antiviral innate immune response. However, the role of GoSTING in the antiviral response remains unclear. Upregulation of some immune-related genes is an important strategy for the host to fight infection. To determine whether GoSTING could respond to the RNA virus NDV, we analyzed the expression of GoSTING, some cytokines, and the ISGs in GEFs following infection with NDV using qRT-PCR. The results illustrated that the mRNA levels of GoSTING in the NDV-infected GEF cells were significantly upregulated during the early stages of infection (**Figure 3A**). The mRNA levels of IFNs (IFN-α, IFN-γ) (**Figures 3B**, **C**), IL-6 (**Figure 3D**), and ISGs (Mx-1 and PKR) (**Figures 3E**, **F**) were significantly upregulated as well. Cells resist the invasion of foreign viruses by upregulating the expression of these genes.

### GoSTING involved in the regulation of IFNs

**Figure 4:**
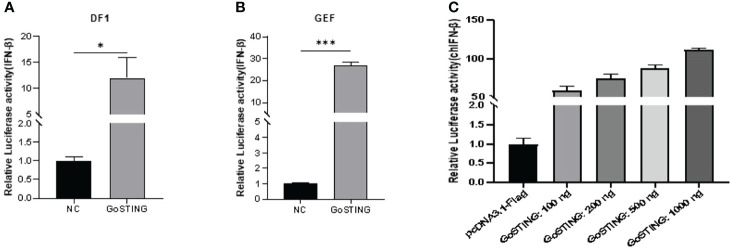
GoSTING is involved in regulating IFN-β. **(A)** DF-1 cells were cotransfected with luciferase reporter plasmids (pRL-TK and pGL-IFN-β-Luc) and pcDNA3.1-GoSTING-Flag or empty plasmid. Luciferase assays were performed after 24 hours of cotransfection. **(B)** GEFs were cotransfected with IFN-β luciferase reporter plasmids and with pcDNA3.1-GoSTING-Flag or pcDNA3.1-Flag. Luciferase assays were performed 24 hours after transfection. **(C)** GoSTING dose-independently induced IFN-β induction. The difference between the experimental and control groups was *p < 0.05 or ***p < 0.001.

STING has been a critical mediator of virus-triggered type I IFN signaling in chicken and duck cells through different pathways (14, 29, 31). To investigate whether GoSTING is also involved in the type I IFN signaling pathway, we transfected DF-1 cells with constructs expressing GoSTING and the empty vector, respectively, and examined the IFN-β activation with a luciferase reporter assay. The results showed that the overexpression of GoSTING resulted in a remarkable activation of the chIFN-β promoter in DF-1 cells (**Figure 4A**), and the activation of IFN-β exhibited a positive correlation with a dosage of the GoSTING plasmid (**Figure 4B**). To further confirm the ability of IFN activation of GoSTING, we prepared primary GEFs. Furthermore, luciferase assays were conducted with GEFs. Similarly, the overexpression of GoSTING in GEFs activated the IFN-β promoter (**Figure 4C**).

### The essential domains of GoSTING in IFN activation

**Figure 5:**
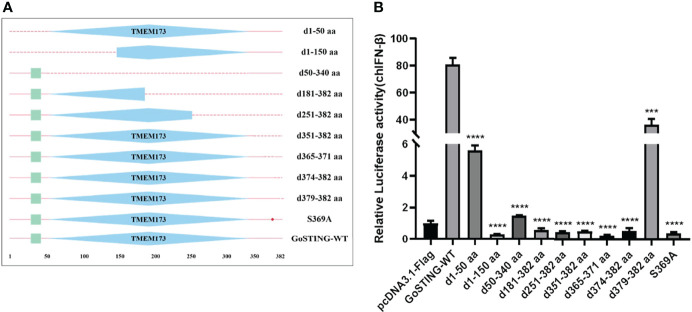
The Essential Domains of GoSTING in IFN Activation. **(A)** Schematic structure of GoSTING mutants. **(B)** The effects of GoSTING truncated mutants on IFN-β promoter activity. Cells were transfected with different expression plasmids of GoSTING and the reporter plasmids pGL-IFN-β-Luc and internal control Renilla luciferase (pRL-TK). Luciferase assays were performed 24h after transfection. All luciferase assays were repeated at least three times, and the difference between the experimental and control groups was ***p<0.001 or ****P<0.0001.

Based on the structural domains of GoSTING predicted by the SMART program, a series of truncated mutants lacking different function domains were constructed (**Figure 5A**). Their ability to activate the IFN-β promoter was assessed with dual-luciferase reporter assays. As shown in **Figure 5B**, the deletion of 50 (d1-50 aa) amino acids showed a significant decrease in the ability to activate IFN-β compared with the wild-type GoSTING. The further deletion of 290 residues in the GoSTING (d50-340aa) resulted in a remarkable decrease in promoter activity. In contrast, for the deletion mutant, GoSTING-d379-382aa, even with a deletion of only 4 aa at the C-terminal, led to such a strong decrease in IFN-β induction. The N-terminal deletion mutant (d1-150aa), the C-terminal deletion mutant (d181-382aa, d251-382aa, d351-382 aa, and d374-382aa) and the mutant deleted 365-371 amino acids (d365-371aa) failed to activate the IFN-β promoter. Moreover, the S369 (corresponds to the S366 in human STING) seems to play a decisive role in IFN activation since the S369A mutant failed to activate IFN-β completely.

### GoSTING plays an important role in anti-RNA viruses infection *in vitro*

**Figure 6:**
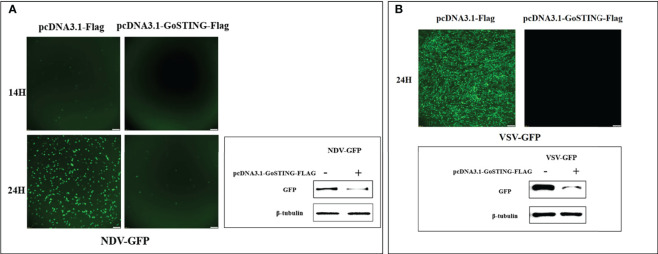
GoSTING inhibits viral yield. **(A)** Viral fluorescence in DF-1 cells transfected with pcDNA3.1-Flag or pcDNA3.1-GoSTING-Flag and infected with NDV-GFP at 0.01 MOI. Error bars represent standard deviations and Western blots for the expression of the NDV-GFP. **(B)** Viral fluorescence in DF-1 cells transfected with pcDNA3.1-Flag or pcDNA3.1-GoSTING-Flag and infected with VSV-GFP at 0.01 MOI. Error bars represent standard deviations and Western blots for the expression of the VSV-GFP.

To test the antiviral effects of GoSTING, the GoSTING-overexpressing and normal DF-1 cells were infected with NDV-GFP and VSV-GFP, respectively, and fluorescence was measured with a fluorescence microscope. The fluorescence intensities of both NDV-GFP and VSV-GFP in GoSTING overexpression cells were significantly lower than those in the control DF-1 cells at 14 and 24 h after viral infection (**Figures 6A, B**). To further investigate the GoSTING’s role during viral infection, the virus-infected cells were then lysed to detect the expression of NDV-GFP and VSV-GFP using Western blot. The protein band results showed that GoSTING could substantially reduce the expression of both NDV-GFP and VSV-GFP (**Figures 6A, B**). These results indicate that the overexpression of the GoSTING in DF-1 cells could inhibit NDV-GFP and VSV-GFP viral replication.

## Discussion

STING is a key signaling molecule that regulates innate immune signaling processes. Previous studies in our laboratory found that overexpression of chSTING and DuSTING in their respective cells could activate the IFN-β promoter and exert antiviral effects. Compared with chickens and ducks, the ability of geese to resist NDV and AIV showed a more significant advantage (35, 36). STING as a key IFN regulator may be one of the reasons for the difference in antiviral ability. A better understanding of the functions of GoSTING may help explain these differences. Currently, the functional characterization of GoSTING is pending and controversial. Therefore, it is necessary to carry out functional research on GoSTING.

In this study, GoSTING was identified with an open reading frame of 1149 bp, encoding 382 amino acid residues (**Figure 1A**). According to the prediction of the SMART website, GoSTING contains a TMEM173 (50–340 aa) domain (**Figure 1B**), which is highly conserved in GoSTING and other mammalian STINGs, indicating its important function in the host. Using MegAlign software alignment, the amino acid sequence of GoSTING was 93.5% similar to that of DuSTING, far exceeding that of other species (range from 32.1% to 69%), even to its closest relative birds, chickens, and zebra finches, the amino acid similarities were only 69% and 61.8% (**Figure 2A**). Similar results can be obtained by phylogenetic tree analysis (**Figure 2B**). The STING protein sequences of geese, ducks, zebra finches and chickens belong to one subgroup. The STING of mammals, including goats, cattle, pigs, cats, chimpanzees, humans, monkeys, and mice, belong to another subgroup. STING sequences from zebrafish belong to a third subgroup. The above results reflect that GoSTING has a closer genetic relationship with poultry, especially ducks. The predicted three-dimensional structures of GoSTING are shown in **Figure 2C**.

For the host, the production of IFN to induce the expression of ISGs, which is a powerful viral restriction factor in establishing an antiviral state, is a common strategy to resist viral infection (3). GEF cells were transfected with GoSTING or empty vector and then infected with NDV. As expected, the qRT-PCR test showed that the virus could significantly upregulate the mRNA levels of IFNs (IFN-α and IFN-γ) (**Figures 3B, C**) and downstream ISGs, including Mx-1 and PKR (**Figures 3E, F**), which have been shown to play an important role in the antiviral innate immune defense of IFN (37, 38). The proinflammatory factor IL-6 showed the same expression trend (**Figure 3C**). Many studies have shown that mammalian STING can act as an IFN-activated gene. To elucidate whether GoSTING has the same function, we overexpressed GoSTING in GEF or DF-1 cells and examined the activity of the IFN-β promoter by a dual-luciferase reporter assay. The results showed that overexpression of GoSTING could strongly activate the IFN-β promoter, and this induction was positively correlated with the dose of transfected GoSTING (**Figures 4A–C**). Based on the above findings, we infer that GoSTING inhibits NDV replication and exerts immunomodulatory effects by activating IFN pathway disorders and inducing some ISGs in the early stage of viral infection.

To identify the GoSTING domains important for IFN induction, a series of truncated forms of GoSTING mutants were generated, and their relative induction of IFN-β promoter activity was measured (**Figure 5**). The results showed that GoSTING mutants’ ability to miss the entire TMEM173 domain to activate the IFN-β promoter was significantly reduced, indicating that the TMEM173 domain was necessary for GoSTING to activate IFN-β, which was consistent with the previous TMEM173 domain of mammalian STING. Subtle changes in the amino acid sequence of TMEM173 affect STING-dependent innate immune signaling by reducing the ability to activate type I IFNs (13). Chen et al. determined that the carboxy-terminal region of STING is required and sufficient for activation of TBK1 and stimulation of IRF3 phosphorylation (39). Deletion of GoSTING C-terminal amino acid fragments of different sizes (d181-382aa, d251-382aa, d351-382aa, d374-382aa, and d379-382aa) resulted in a marked reduction in their ability to activate the IFN-β promoter. GoSTING-d1-50aa and GoSTING-d1-150aa, lacking 50 and 150 amino acids at the N-terminus of GoSTING, respectively, also had significantly reduced activation ability compared with wild-type GoSTING, which we speculate may be due to a barrier in its localization to the organelle. The underlying mechanism of STING regulation is phosphorylation at some sites in response to stimulation of cytoplasmic DNA (39). Our results show that the S369A point mutant abolished its IFN-β activation, suggesting that serine 369 may be an important serine site for STING activation.

Recently, the role of STING in inhibiting RNA viruses has attracted increasing research interest. RNA virus can activate STING and upregulate its expression after invading the host (40, 41). Deletion of STING renders murine embryonic fibroblasts (STING^-/-^MEFs) highly susceptible to infection by minus-strand viruses, including vesicular stomatitis virus (VSV) (42). ChSTING exhibits antiviral function against RNA viruses NDV and VSV (29, 43). We performed a series of experiments to clarify whether GoSTING also has antiviral activity. By monitoring the GoSTING mRNA level, we found that NDV can regulate GoSTING at the transcriptional level and upregulate the expression of GoSTING, and this phenomenon is particularly evident in the early and middle stages of virus infection, which indicates that GoSTING may play an important role in NDV infection. We thus explored the effect of GoSTING on viral replication. The results showed that GoSTING overexpression in DF-1 cells significantly inhibited the viral replication of NDV-GFP and VSV-GFP (**Figure 6**). In fact, research on STING for confinement of DNA viruses has already started (44). STING has been reported to be involved in innate immune defenses triggered by adenovirus, herpes simplex virus, and papilloma virus (45–47). Studies on chickens and ducks have also shown that in addition to RNA viruses, both chSTING and DuSTING show resistance to DNA viruses (14, 48, 49). Based on the conservation of amino acids in the STING protein that are critical for the recognition of various exogenous nucleic acid moieties (30) and the close kinship of geese to two other avian species, we speculate that GoSTING is required for host responses to both DNA and RNA viruses. This paper demonstrates that GoSTING plays an important role in RNA virus infection, but the role of GoSTING in DNA virus needs further study.

Nowadays some progress has been made in the research of STING in the innate immunity of birds. In this study, the amino acid sequence alignment showed that the homology of GoSTING to DuSTNG and chSTING was 93.5% and 69.0%, respectively (**Figure 2A**). It can be concluded that GoSTING has high homology with STING of birds, especially ducks. In addition, the results of protein domain prediction showed that the TMEM173 domain is conserved in birds, suggesting that birds may be similar in the activation of STING and the recognition of PAMPs. Previous studies have shown that overexpression of chSTING in DF-1 cells can significantly inhibit the replication of AIV and NDV, accompanied by an increase in pro-inflammatory cytokines such as IFN-β, IL-1β, and IL-2 (29). The effect of DuSTING on IFN activation and anti-RNA virus has also been elucidated by multiple investigators (31, 50). This study determined the functional characterization of GoSTING and found that GoSTING also has similar functions. We therefore conclude that STING is an essential IFN mediator that plays a role in avian innate immunity against RNA viruses. It is worth mentioning that both chSTING and DuSTING show resistance to DNA viruses, but whether GoSTING has the ability to resist DNA viruses remains to be studied.

However, the IFN signaling mechanisms of STING in chickens, ducks, and geese may be different, although they belong to the same bird species. At present, a relatively comprehensive study of the innate immune signaling pathway in chickens has been carried out. The biggest difference between the RLR pathway of chickens, ducks and geese is that chickens lack RIG-I (51), a key receptor for sensing many RNA viruses in birds, including AIV and NDV (32), making chickens more susceptible to some viruses, especially RNA viruses that require RIG-I for recognition. Previous studies in our laboratory showed that chSTING senses AIV virus by using MDA5 to compensate for RIG-I, and conducts signal transduction through MDA5-STING-IFN pathway (29). Nonetheless, chicken MDA5 is not sufficient against AIV, and AIV often causes lethal death in chickens (30). In contrast, ducks and geese tend to be natural hosts for many asymptomatic AIV subtypes (30), which are related to the molecular basis of their RIG-I. DuSTING was identified as an important receptor that responds to AIV infection and induces IFN-β production, but how RIG-I works for the function of DuSTING in RIG-I present ducks is unclear (31). Our results suggest that GoSTING is an important regulator of IFN, pro inflammatory cytokines and ISGs, and plays a role in antiviral innate immunity in geese. However, the current research on the RLR pathway of waterfowl, especially geese, is still relatively fragmented. Although both GoRIG-I (32) and GoMDA5 (52) have been shown to play a role in the anti-RNA virus innate immunity of geese, whether pathogen-associated RNA triggers STING signaling through RIG-I or MDA5 or whether RIG-I and MDA5 share the downstream STING signaling pathway is unclear. Future experiments based on RIG-I or MDA5 knockout duck cell lines may be required for further validation.

To sum up, our findings suggest that GoSTING is an important innate immune modulatory molecule involved in antiviral innate immunity in geese through its involvement in the type I IFN signaling pathway. The overexpression of GoSTING can upregulate several important pivotal ISGs and proinflammatory factors and combat NDV infection. Our study complements the functional characteristics of GoSTING, enriches the overall understanding of avian STING, and contributes to a more comprehensive and systematic understanding of the anti-RNA virus innate immune signaling pathway of avian STING.

## Data availability statement

The original contributions presented in the study are included in the article/supplementary material. Further inquiries can be directed to the corresponding authors.

## Ethics statement

The studies involving goose embryos were conducted in the laboratory of Shanghai Veterinary Research Institute. The studies were reviewed and approved by the Animal Ethics Committee of Shanghai Veterinary Research Institute (20210521). The animal study was reviewed and approved by the Animal Ethics Committee of Shanghai Veterinary Research Institute.

## Author contributions

YC and JS designed the experiment. FF and ZL performed the majority of the experiments. JW, PL, YanL and YawL helped with the experiments. FF, ZL and YC wrote the paper. ZW, JM, and YY helped analyze the experimental results. All authors contributed to the article and approved the submitted version. All authors contributed to the article and approved the submitted version.

## Funding

This research was supported by the Natural Science foundation of Shanghai (20ZR1425100), the National Natural Science Foundation of China (32072865, and 32072864), Science and Technology Commission of Shanghai Municipality (21N41900100), the Interdisciplinary Program of Shanghai Jiao Tong University (YG2021QN108), and State Key Laboratory of Veterinary Biotechnology Foundation Grant (SKLVBF202107).

## Conflict of interest

The authors declare that the research was conducted in the absence of any commercial or financial relationships that could be construed as a potential conflict of interest.

## Publisher’s note

All claims expressed in this article are solely those of the authors and do not necessarily represent those of their affiliated organizations, or those of the publisher, the editors and the reviewers. Any product that may be evaluated in this article, or claim that may be made by its manufacturer, is not guaranteed or endorsed by the publisher.
